# Validation of a force transducer-embedded platform as an alternative to handles in weight perception research

**DOI:** 10.3758/s13428-025-02824-x

**Published:** 2025-10-21

**Authors:** J. W. C. Harris, M. J. Murphy, P. A. Chouinard

**Affiliations:** Department of Psychology, Counselling, and Therapy, School of Psychology and Public Health, La Trobe University, Bundoora Campus, Melbourne, VIC Australia

**Keywords:** Weight perception, Methodology, Lifting forces, Validation, Size–weight illusion, Sensorimotor processes

## Abstract

The recording and interpretation of lifting force data — such as load and grip forces — are central to studying weight perception. Typically, such data are collected using force transducer-embedded handles placed on top of objects. While effective, these handles may be impractical or undesirable for certain experimental paradigms. A potential alternative is a force transducer-embedded platform, but validation is needed to determine whether it captures force data with the same consistency and interpretability as the handle-based method, particularly given the potential for data loss around lift-off. In two experiments, we compared these methods by having participants lift light and heavy objects off a platform either directly or via handles to assess the convergent validity of experimental outcomes and the concurrent validity of the recorded data. Our findings indicate that the experimental outcomes and data from both methods were highly comparable, but only for the heavy objects. However, for the light object, platform-recorded force data showed lower agreement with handle-based measures, and several anticipated sensorimotor effects were not observed in the platform data. These discrepancies resulted in differences in experimental outcomes, particularly in the detection of switch effects, highlighting the platform’s limitations for capturing lighter-weight interactions. Therefore, we suggest that while handles remain preferable for capturing rich force data, the platform method broadens methodological options and presents a viable and valid alternative.

## Introduction

The forces we exert when grasping and lifting objects are important to understanding weight perception and the size-weight illusion (SWI) — a phenomenon where the smaller of two equally weighted objects is perceived as heavier (Charpentier, 1886, 1891). These forces have been used as a proxy measure of our a priori, or implicit, expectations of the weight of objects, whereby greater initial application of force relates to a greater expectation of the object’s mass (Chouinard et al., 2005). Force data have been instrumental not only in exploring whether force application itself influences weight perception or, alternatively, adapts over time (e.g., Davis, 1973; Flanagan & Beltzner, 2000; Grandy & Westwood, 2006; Granit, 1972), but also in practical aspects, for example, in confirming participants’ adherence to task instructions, such as lifting stimuli in a straight, vertical path to avoid confounding variables related to torque (Flanagan & Beltzner, 2000). Given how widespread and influential force data have become in the study of weight perception and related phenomena, how these data are acquired and how different methods compare are important to research outcomes and therefore warrant careful consideration.

In many studies, force data are obtained through six-axis force–torque transducers embedded in a handle, which is placed on top of the stimulus and gripped between the thumb and index finger in a pinching motion to lift the object (Chang et al., 2008; Chouinard et al., 2009; Engel et al., 2020; Flanagan & Beltzner, 2000; Green et al., 2010; Mon-Williams & Murray, 2000). These transducers record the forces and torques applied across three dimensions from which two main types of data are typically derived: grip force, the horizontal force applied to stabilise the object, and load force, the vertical force applied to overcome the object’s mass during lifting. While this is an effective, standard method for capturing force data, it is not without limitations. For example, one key constraint of handle-based setups is that they require a precision grip, limiting how participants can interact with the object. This restriction narrows the generalisability of findings to more naturalistic forms of object interaction — such as enclosed grips — which are common in everyday contexts.

There are also logistical drawbacks. If researchers want to use a large number or range of stimuli, it may require investing in a corresponding number of transducers, which could quickly become expensive. While transducers can be exchanged between objects in between trials, this process can disrupt the experimental flow, increase the duration between trials, and reduce the overall number of trials that can be conducted within a given timeframe. Moreover, this exchange introduces the possibility of experimental error — for example, inconsistent incorrect reconnection — which could otherwise be avoided. Each of these issues may be minimised when using a platform with a fixed transducer, as no exchange between stimuli is required, though care must still be taken to ensure objects are placed centrally. Although the frequency of such errors is difficult to determine — given they are rarely reported in detail — their potential impact on data quality, coupled with the reduction in experimental burden, may justify this trade-off in studies where grip force data are not essential.

However, an alternative approach has been utilised in which force transducers are embedded within a platform from which stimuli are lifted (Baugh et al., 2016; Flanagan et al., 2008; Trewartha & Flanagan, 2017). As objects are lifted from the platform, the transducers detect a reduction in the downward force exerted on the platform, corresponding to the object’s weight being removed during lift. Unlike the handles, this method allows for greater flexibility in lifting behaviours and handling strategies, broadening the scope of potential applications. However, as with the handles, the platform method has its own limitations.

One clear difference between the handles and the platform is that the former allows the collection of grip force data, while the latter does not. Grip force measures are prevalent in the literature, with up to two-thirds of SWI studies incorporating some form of grip force data (Harris et al., 2024). However, some studies focus solely on load force, as it directly reflects the vertical force applied to counteract gravity and lift the object, and is therefore often considered a more direct indicator of participants’ sensorimotor predictions regarding object weight (Flanagan et al., 2001, 2008). When available, grip force data are often combined with load force data to assess both expectations of object mass (e.g., anticipatory grip force scaling) and adaptation to that mass (e.g., Buckingham & Goodale, 2010; Flanagan & Beltzner, 2000). As such, grip force data serve two important purposes. Firstly, they allow for a more nuanced examination of the sensorimotor processes involved in object lifting, for example, by providing additional information such as object stabilisation. Secondly, they offer convergent evidence when combined with load force data, which can enhance confidence in the conclusions drawn. Without access to measures like grip force, interpretations may become more limited or less precise. Nevertheless, the platform setup enables the collection of load force data during lifts involving a broader range of grip types — interactions that would otherwise preclude any force measurement in handle-based designs.

**Fig. 1:**
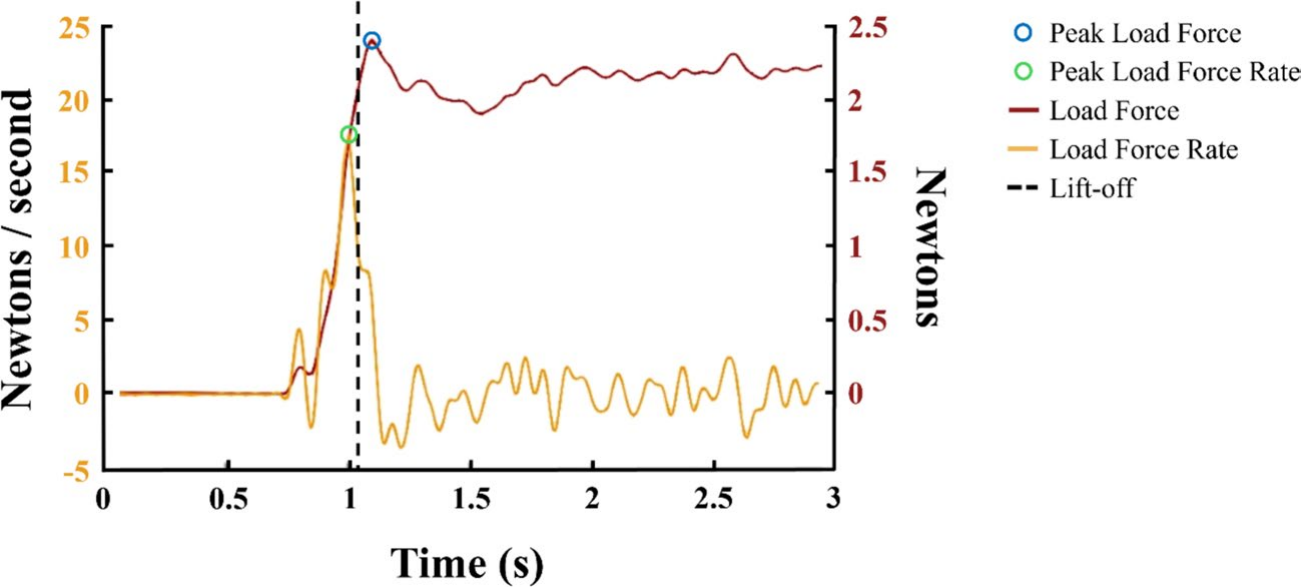
Illustrative example of load force and force rate data. The red line represents load force (in newtons; right axis), and the yellow line represents load force rate (in newtons/second; left axis). The peak load force rate (green circle) may occur just before lift-off (dashed line), making it more likely to be captured by the platform. However, the peak load force (blue circle) may occur just after lift-off, which could prevent the platform from fully recording it. *Note:* the figure is adapted with permission from Saccone et al. (2019)

Since the platform-based method captures only load forces, ensuring that the quality and interpretability of these data are comparable to those produced by handles becomes especially important. One could reasonably assume that an application of load force to an object would be paired with an equivalent decrease in load force measure by the platform, but the extent of this agreement is unclear. For example, there may also be data loss with the platform-based method, as it can only measure force data up to the point of lift-off. In some cases—such as when objects are lighter than anticipated — peak force application may occur beyond the point of lift-off —outside of the platform’s recording window. While peak load force rate tends to occur slightly earlier and may still be captured, its proximity to lift-off increases the risk that it is only partially recorded, making the identification of the true peak less precise (Fig. 1; Baugh et al., 2016; Harris et al., 2024; Saccone et al., 2019; Trewartha & Flanagan, 2017).

However, ‘peak’ measures (i.e., peak force and peak force rate) are typically not used in platform-based studies, which instead opt for ‘first peak’ measures which identify the force at the first peak in force rate — in other words, the force which is applied to the object at the point at which there is the greatest change in that applied force (Baugh et al., 2016; Flanagan et al., 2008; Trewartha & Flanagan, 2017). While both peak and first peak measures have been used to evaluate sensorimotor predictions (e.g., Baugh et al., 2016; Buckingham & Goodale, 2010; Flanagan et al., 2008; Harris et al., 2024), they can index different aspects of processing.

To explain, depending on how these measures are selected, they may come from the initial application of force, before adjustments occur, or after, capturing some degree of alterations to the initial force selection. In a practical sense, they provide insight into overall force application in object interactions and how that relates to weight perception (Flanagan & Beltzner, 2000). In comparison, the force at first peak measures are thought of as being an index of the initial prediction of the object’s weight, focusing on how forces are applied before recalibration due to sensorimotor feedback (Baugh et al., 2016; Flanagan et al., 2008). In doing so, the measure isolates the anticipatory response from corrective actions, giving a picture of the subject’s initial weight estimation. As these measures have different implications for the sensorimotor process and the outcome interpretation, and depend upon different durations of data capture, it is also important to evaluate how the platform compares to the handles in acquiring both forms of data.

Given the value of force data in weight perception experiments, the need arises for an alternative collection method when the force–transducer handle is impractical or undesirable. However, it remains necessary to determine how it compares to the more established handle-based method. Therefore, we aimed to assess the validity of a force transducer-embedded platform as an alternative to the traditional handle. Specifically, we compared the platform against the handle in terms of concurrent validity — the degree to which the platform’s load force data aligned with that recorded by the handle — and convergent validity — whether the platform, when objects are lifted directly from it without a handle, reproduced experimental outcomes consistent with those observed using the handle-based method.

To achieve this, we conducted two experiments in which objects were lifted either via a handle attached to the platform (Experiment 1) or directly by hand from the platform (Experiment 2). In Experiment 1, data were recorded simultaneously from both devices, allowing for a direct comparison of the force data captured by the handle and the platform. In Experiment 2, participants lifted the same objects directly from the platform, without handles, allowing us to assess whether outcomes differed as a result of the lifting method. Comparing the results across experiments therefore enabled us to evaluate whether the way the platform is used—either via handle or direct lift—impacts the perceptual and sensorimotor outcomes it produces.

Each experiment involved two manipulations: object weight (light or heavy) and the order of presentation, in which stimuli were either switched (lifting heavy-after-light or light-after-heavy) or not switched (lifting light-after-light or heavy-after-heavy). These manipulations are known to elicit robust and highly replicable sensorimotor effects, where less force is applied when lifting a heavy object after a light one, resulting in the heavy object being perceived as heavier, and vice versa (Chouinard et al., 2005; Harris et al., 2024; Maiello et al., 2018; van Polanen & Davare, 2015). These well-established effects provided a reliable and sensitive basis for evaluating the platform.

## Methods

### Participants

A sample of 29 individuals (7 male, 22 female; mean age = 25.6 years, range = 18 to 42 years) participated in the study and were compensated with gift cards. All participants gave informed, written consent to participate and reported that they were not taking any medications that made them drowsy, and were free of psychological, neurological, and psychiatric disorders. These exclusion criteria were in place such that our sample reflected healthy brain functioning. All participants were right-handed, as confirmed by the Flinders Handedness Survey (Nicholls et al., 2013), and had normal or corrected-to-normal vision, as verified by performance on the Snellen chart and the Random Dot 3 s Stereo Acuity Test (Vision Assessment Corporation, IL, USA).

### Stimuli

**Fig. 2:**
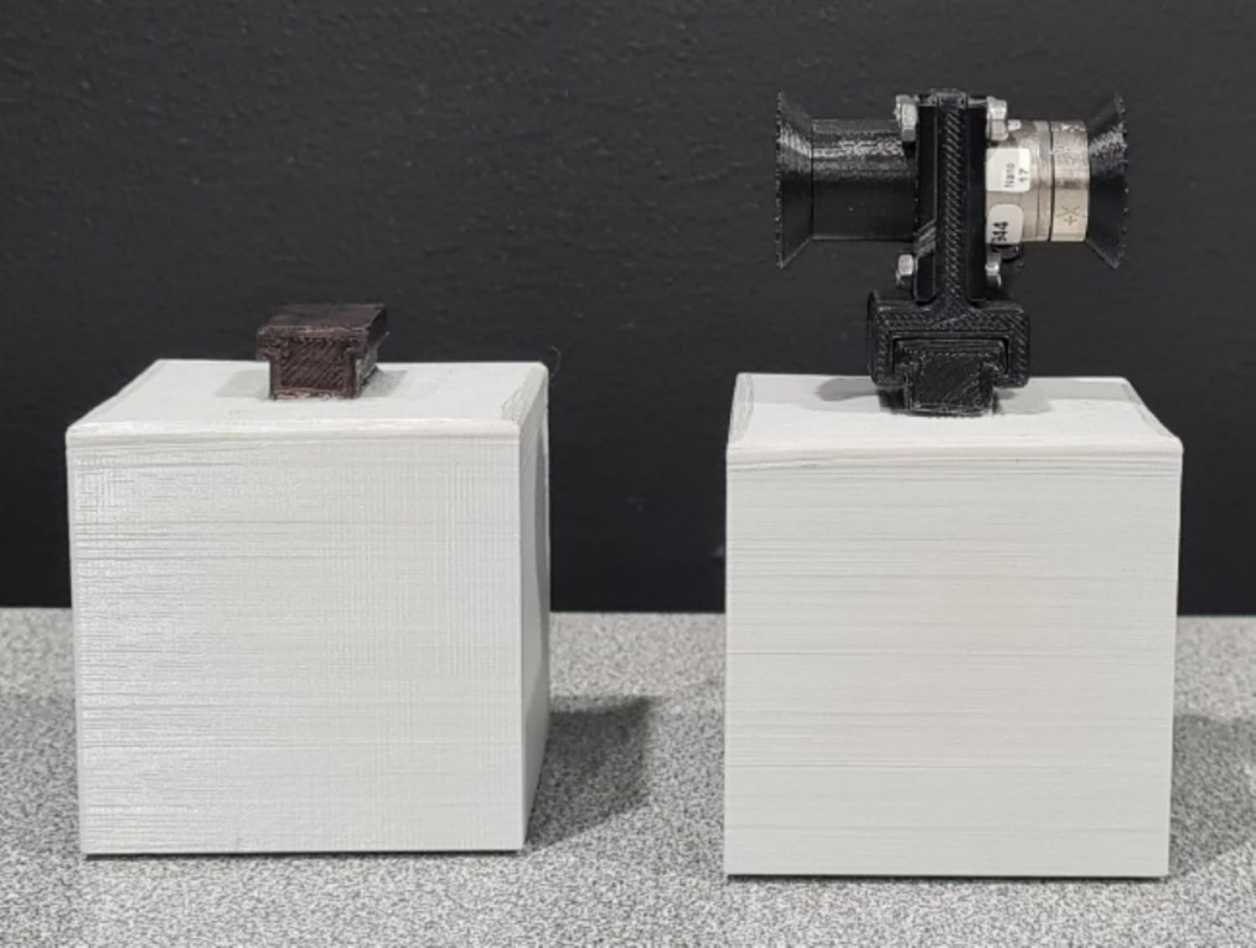
Experimental stimuli: The same two stimuli were used in both the handles and no-handles conditions. The force transducer handle (**right**) was removed from the stimuli for the no-handles condition (**left**)

We used two three-dimensional (3D)printed hollow cubes, both grey and measuring 6 cm × 6 cm × 6 cm, with a volume of 216 cm^3^ (see Fig. 2). A small clip was affixed to the middle of the top surface of each cube, allowing the force transducer handles to be placed securely. The cubes were filled with lead shot, resulting in weights of 125 g (light) and 518 g (heavy), with densities of 0.58 g/cm^3^ and 2.40 g/cm^3^, respectively. Care was taken to place the lead shot in the geometric centre of the objects to reduce the influence that mass distribution can have on the perceived weight of objects (Amazeen & Turvey, 1996; Harris & Chouinard, 2023).

### Apparatus

Force data were recorded at a rate of 400 Hz using Nano17 F/T force transducers (ATI Industrial Automation, Garner, NC, USA) attached to both the handles of the cubes and the platform. The transducers on the handles measured force and torque exerted in the *x*, *y*, and *z* axes by the index finger during lifting. It should be noted that the force recorded by the handle represents half of the total applied force, as that exerted by the thumb was not measured.

**Fig. 3:**
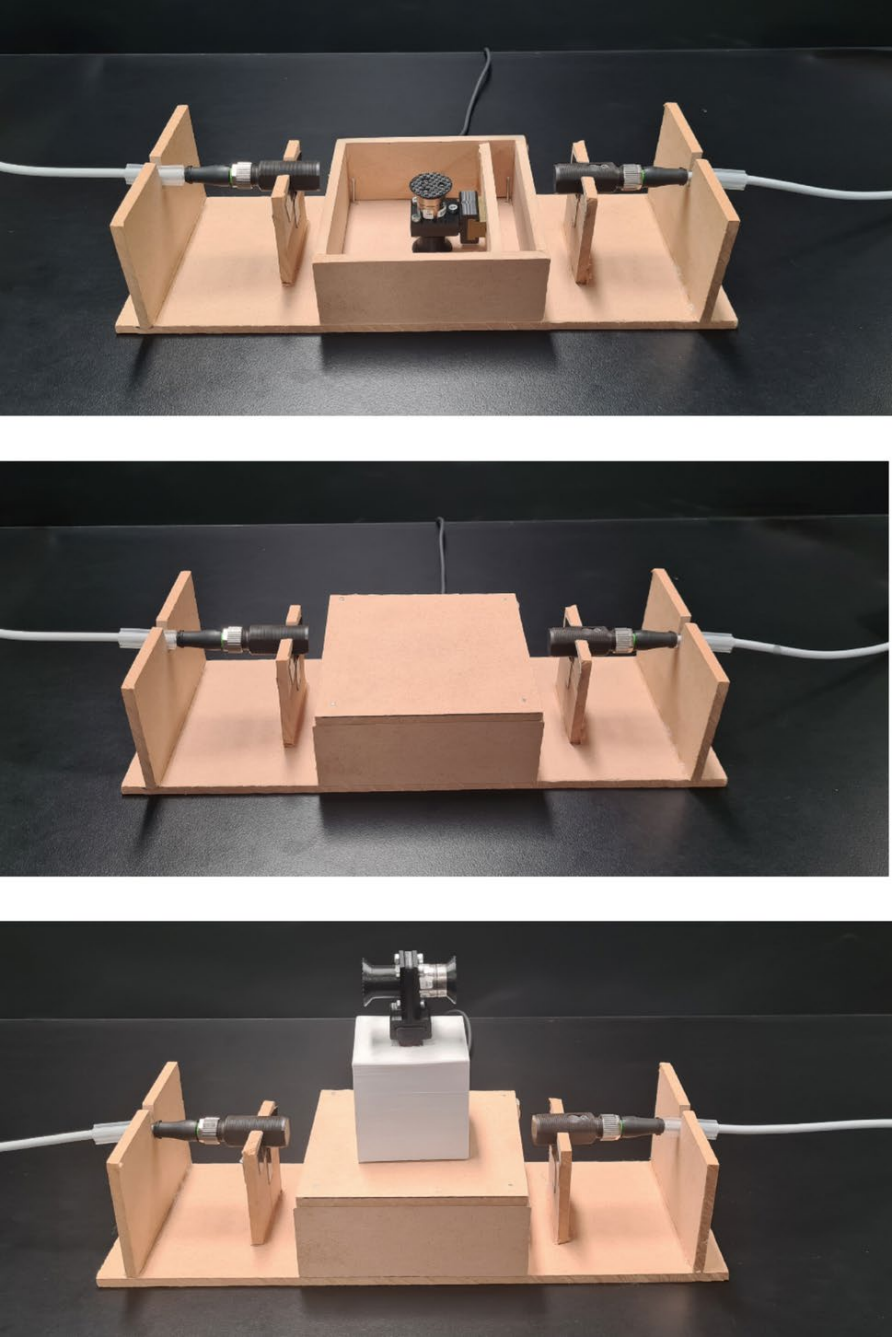
Transducer-embedded platform. The top panel depicts the platform with the lid removed, while the middle panel depicts the platform with the lid in place, and the bottom panel the platform with a stimulus placed on top. Embedded in the platform was a force transducer which measured the decrease in force as objects were lifted from the surface. On the sides are light sensors which signalled when the object left the surface of the platform

As can be seen in Fig. 3, a wooden box measuring 10 cm × 10 cm × 6 cm served as the base of the platform. Within the platform base, a short wooden slat was fixed in place, onto which a peg was mounted. This was positioned such that, when the force transducer was clipped to the peg, it sat securely at the centre of the platform. The transducer was oriented with its *z*-axis facing vertically — that is, aligned with the direction of applied force as the object was lifted. Resting on top of both the platform base and the transducer was a 10 cm × 10 cm × 0.6 cm wooden lid. To prevent lateral displacement of the lid when objects were placed there, small nails were driven into each corner about 0.8 cm diagonally inward. These nails did not contact the platform walls or base, and did not hold the lid down and therefore had no influence on the recorded force data; they simply acted as stops against sideways shifting. Care was taken to ensure that the platform and the lid were level and that the base sat flush against the supporting surface. Small adhesive foam pads were affixed to the underside corners of the lid; these were sized and positioned such that they did not bear any of the object’s weight or interfere with the force readings during lifting, as confirmed during prototyping of the platform. They were included solely as a precautionary measure to protect the transducer from potential damage if objects were placed forcefully or off-centre.

To allow for precise detection of object lift-off, light sensors were positioned on either side of the platform. These sensors were mounted to thin wooden slats, each with a cut-out section enabling the sensors to sit just above the surface of the lid. The sensors were positioned approximately 2 cm from the edge of the platform to ensure that participants did not inadvertently strike them when placing objects back down.

It should also be noted that the platform used here represents one possible construction of a force transducer-embedded platform. Other platform designs may differ in their mechanical characteristics, and certain considerations may not apply — or may apply differently — to alternative constructions. For example, if the lid were attached directly to the transducer, potential considerations related to lid movement might be mitigated, though this approach could introduce practical challenges, particularly given the delicate nature of the equipment. Additionally, in our design, the lid was constructed from wood, and it is theoretically possible that the material properties — such as density, stiffness, and vibration damping — differ from those of other materials, such as metal. While any such influence would likely be more relevant to measurements involving force on object placement rather than lift-off, it nonetheless represents a possible setup-specific factor for consideration.

### Procedure

Participants were randomly assigned to start with either Experiment 1 or 2, to control for order effects. Each experiment consisted of 81 lifts where stimuli were presented in a predetermined, pseudo-random order, ensuring 20 trials for each cube and switch condition. In other words, there were 20 light-after-light, 20 light-after-heavy, 20 heavy-after-heavy, and 20 heavy-after-light trials. The first trial of each experiment was discarded, as it did not involve either a switch or a repeated trial with the same mass, and was simply the starting trial, leaving 80 trials for analysis per experiment.

Participants wore a pair of PLATO Visual Occlusion Spectacles (Translucent Technologies Inc., Toronto, ON, Canada), which remained closed throughout the experiment, only opening during a trial. This blinding procedure ensured that participants’ responses to the objects were not influenced by observing the experimenter interacting with the stimuli (Buckingham et al., 2014; Harris et al., 2024, 2025; Saccone et al., 2019), safeguarding against experimenter effects (Firestone & Scholl, 2016; Harris et al., 2024). Additionally, the stimulus not currently being presented to the participant was hidden behind a screen so as to remain out of their view when the spectacles opened. A trial began when the spectacles opened, signalling to the participant to grasp the stimulus and lift it approximately 5 cm off the platform in a smooth, confident manner. Participants kept the object suspended until a beep sounded, indicating the end of the trial, at which point the spectacles closed, 4 s after opening. Participants then placed the object back onto the platform and provided a perceptual estimation of its weight using absolute magnitude estimations (Zwislocki & Goodman, 1980). Specifically, participants gave a numerical estimation of the object based on perceived, rather than expected, heaviness, where greater numbers indicated a heavier object. There were no restrictions on the scale participants used for their magnitude estimates. However, participants were instructed to maintain consistency with their selected scale throughout the experiment.

### Data preparation

Participant perceptual magnitude estimates were normalised by converting them into percent (*%*)-scores. Specifically, for each participant, we subtracted the individual score from their total estimate mean, then divided that value by the total estimate mean before multiplying the result by 100. These %-scores were used in the analysis below for both experiments.

Force recordings were smoothed using a fourth-order, zero-phase lag, low-pass Butterworth filter, with a cut-off frequency of 14 Hz, prior to analysis (Chouinard et al., 2009; Flanagan & Beltzner, 2000). From these force data, several specific measures were acquired: peak grip force (PGF), which described the maximum force applied horizontally to the force transducer, and peak load force (PLF), which described the maximum vertical lifting force applied. To calculate these values, the force data were plotted over time, and the peaks were visually identified and manually selected by a rater. To reduce human error, the value of each true maximum near the selected peak was recorded using an in-house MATLAB script (Saccone et al., 2019), a procedure which has been found to have excellent inter-rater reliability (Harris et al., 2024; Saccone et al., 2019). Additionally, we determined the rate at which these forces were applied, measured as the peak grip force rate (PGFR) and peak load force rate (PLFR). This was done by differentiating each force signal using a three-point central difference equation, which calculated the rate of force application in newtons/second at each time point, with the greatest value being selected. The same method was used to determine the peak platform force rate (PPFR), which described the maximum rate at which force was removed from the platform transducer.

In addition to the peak force measures, we examined load force at the first peak in load force rate (LF1_st_) and platform force at the first peak in platform force rate (PF1_st_). To obtain these measures, we visually identified the first peak occurring within the load phase from the force data plotted over time. Our custom MATLAB script then identified the precise maximum value of the first peak and the corresponding time, subsequently extracting the load force rate at that time.

Finally, we used two different automatic methods to identify the load phase duration — the period between when forces were first applied and when the object was hefted off the platform. The first was the handle-based measure (LPD1). This measure was determined by subtracting the lift-off time, as determined by our light sensors, from the time at which participants first applied more than 0.2 N of load force to the object. The second was the platform-based measure (LPD2) and was calculated in a similar manner: the lift-off time (via light sensors) was subtracted from the time at which more than 0.4 N of force reduction (as, unlike the handles, the platform measured full force, not half) was detected by the force transducer in the platform. For all variables, after initial processing was completed, we removed any data point from analysis which was greater than ±3 standard deviations from the mean. In total, 384 data points were removed, representing 0.01% of the dataset.

### Data analysis

Statistical analyses were conducted using JASP (version 0.16.4, University of Amsterdam, Amsterdam, Netherlands) and GraphPad Prism (version 9.1, GraphPad Software, San Diego, CA, USA). All tests were two-tailed.

Similar 2 (Cube: light vs heavy) × 2 (Switch: switch vs no-switch) repeated-measures analyses of variance (ANOVAs) were conducted for each dependent variable in Experiment 1 (%-scores, PLFR, PLF, PGFR, PGF, LF_1st_, LPD1, PPFR, PF_1st_, and LPD2) and Experiment 2 (%-scores, PPFR, PF_1st_, and LPD2) to assess the convergent validity of the platform-based method relative to the handle-based method. For interaction effects, we report only the comparisons between switch and no-switch conditions for the heavy and light objects, as other comparisons were not of interest. Where appropriate, Bonferroni family-wise corrections were applied to the *p*-values to account for multiple comparisons, and effect sizes are reported as Cohen’s *d*.

To examine the concurrent validity of peak force (PLFR and PPFR), force at first peak (LF_1st_ and PF_1st_), and load phase duration (LPD1 and LPD2) data collected via the handles and platform in Experiment 1, we conducted a series of intraclass correlations (ICC; McGraw & Wong, 1996). ICCs are commonly used to assess the degree of agreement between different measures of the same quantity, with values ranging from 0 to 1 (higher values indicating stronger agreement). We used ICC form (3, 1), which is appropriate when comparing fixed measurement methods — in this case, the handle and platform — and when there is no intention to generalise beyond these specific instruments (Koo & Li, 2016). The ICC values were interpreted according to Koo and Li (2016), where < .50 indicates poor agreement, .50 – .75 indicates moderate agreement, .75 – .90 indicates good agreement, and > .90 indicates excellent agreement.

## Results

### Experiment 1: Lifting objects with handles

**Fig. 4:**
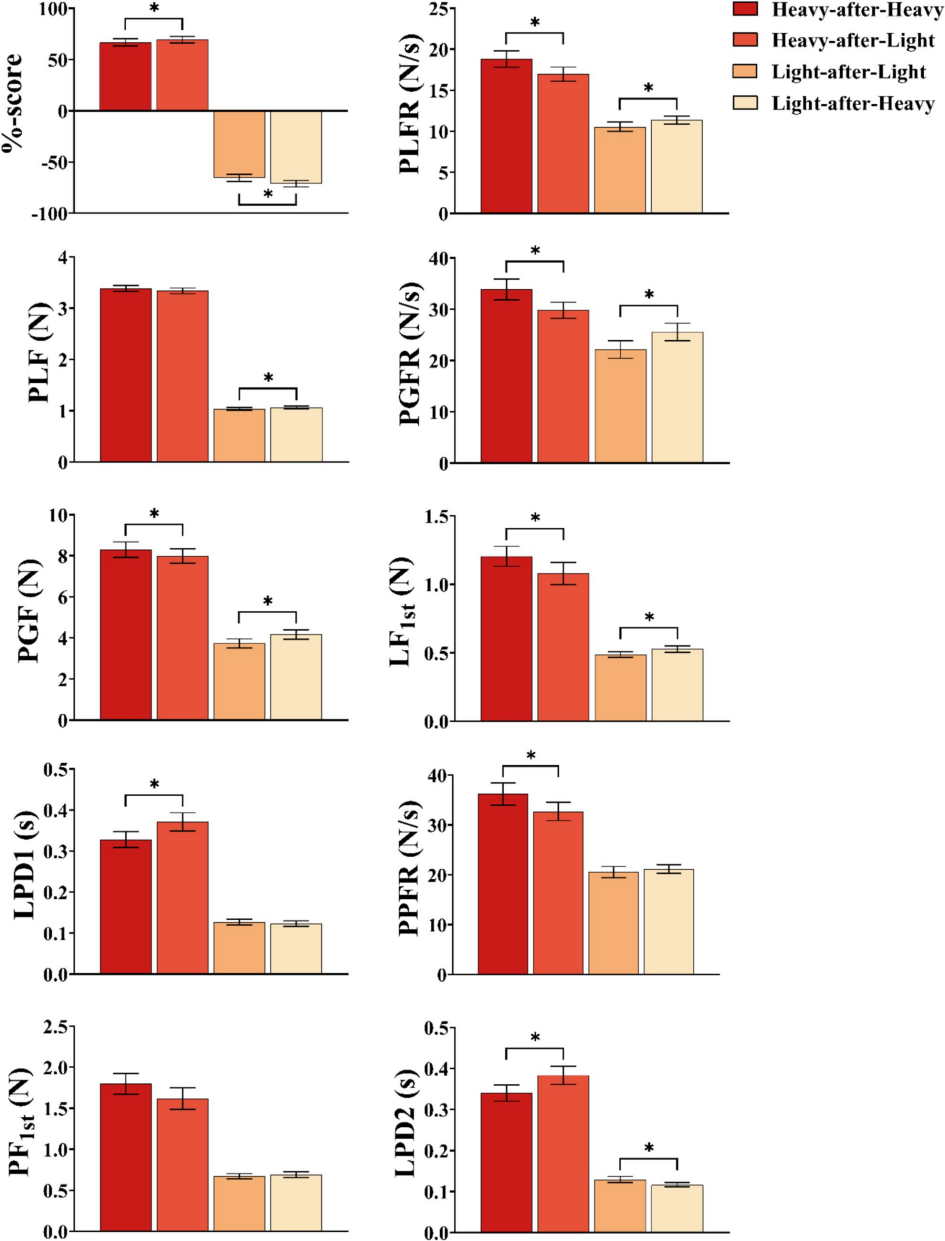
ANOVA results for dependent variables in Experiment 1. Displayed are means with error bars representing ±1 standard error of the mean for %-scores, peak load force rate (PLFR), peak load force (PLF), peak grip force rate (PGFR), peak grip force (PGF), load force at first peak in load force rate (LF_1st_), load phase duration 1 (LPD1), peak platform force rate (PPFR), platform force at first peak in platform force rate (PF_1st_), and load phase duration 2 (LPD2). Only comparisons of interest are displayed (i.e., switch vs no-switch for the heavy and light cubes). Asterisks (*) denote a significant effect (*p* < .05). N = newtons, s = seconds

Overall, for the handle-based measures, we found strong and consistent interaction effects across perception, PLFR, PLF, PGFR, PGF, LF_1st_, and LPD1, where the heavy cube was perceived as lighter and lifted with less force when it followed a light cube, and the light cube was perceived as heavier and lifted with more force when it followed a heavy cube. In contrast, the platform-based measures revealed interaction effects only for the heavy cube in PPFR, for both in LPD2, and for neither in PF_1st_. A summary of the ANOVA outcomes is provided in Table 2 (which presents ANOVA outcomes for both Experiments 1 and 2) with individual variables presented in Fig. 4.

#### Perception (%-scores)

The ANOVA on participants’ mean perceptual %-scores revealed an expected main effect of cube (*F*(1, 28) = 438.91, *p* < .001, *η*_*p*_^*2*^ = .94), in which the heavy cube was perceived to be heavier than the light cube (mean difference = 136.37, *t* = 20.95, *d* = 7.62, *p* < .001). There was a Cube × Switch interaction (*F*(1, 28) = 16.59, *p* < .001, *η*_*p*_^*2*^ = .37) in which the heavy cube was perceived as being heavier when lifted following a light versus heavy cube (mean difference = 2.66, *t* = 2.07, *p* = .047; Fig. 4). A similar effect was found for the light cube, which was perceived as being lighter when lifted following a heavy versus light cube (mean difference = 5.69, *t* = 4.62, *p* < .001).

#### Peak load force rate (PLFR)

There was a main effect of cube (*F*(1, 28) = 177.64, *p* < .001, *η*_*p*_^*2*^ = .86), in which the peak load force rate applied to lift the heavy cube was greater than that applied to lift the light cube (mean difference = 6.93, *t* = 13.33, *d* = 1.69, *p* < .001). There was also a Cube × Switch interaction (*F*(1, 28) = 30.07, *p* < .001, *η*_*p*_^*2*^ = .52; Fig. 4) in which the average peak load force rate applied to lift the heavy cube was lower in the switch (heavy-after-light) as compared to no-switch condition (heavy-after-heavy; 1.85, *t* = 4.72, *d* = 0.45,* p* < .001), and the average peak load force rates for the light cube were greater in the switch (light-after-heavy) as compared to no-switch condition (light-after-light; mean difference = 0.82, *t* = 3.54, *d* = 0.20, *p* = .001).

#### Peak load force (PLF)

There was a main effect of cube (*F*(1, 28) = 2,260.87, *p* < .001, *η*_*p*_^*2*^ = .99), where the heavy cube was lifted, on average, with a greater peak load force than the light cube (mean difference = 2.31, *t* = 47.55, *d* = 9.60, *p* < .001). A Cube × Switch interaction was also found (*F*(1, 28) = 6.77. *p* = .015, *η*_*p*_^*2*^ = .20; Fig. 4), with greater peak load force being applied to the light object when it was lifted following the heavy as compared to light object (mean difference = 0.03, *t* = 2.53, *d* = 0.12, *p* = .018), an effect not seen for the heavy object (*p* = .06).

#### Peak grip force rate (PGFR)

There was a main effect of cube (*F*(1, 28) = 87.81, *p* < .001, *η*_*p*_^*2*^ = .76), where the heavy cube was lifted with a greater average peak grip force rate than the light cube (mean difference = 7.97, *t* = 9.27, *d* = 0.84, *p* < .001). There was also a Cube × Switch interaction (*F*(1, 28) = 30.82, *p* < .001, *η*_*p*_^*2*^ = .52; Fig. 4), with the heavy cube being lifted with a lower average peak grip force rate in the switch (heavy-after-light) as compared to no-switch (heavy-after-heavy) condition (mean difference = 4.07, *t* = 4.09, *d* = 0.43, *p* = .002). The light cube was also lifted with greater peak grip force rate when it was lifted following the heavy object in the switch (light-after-heavy) versus the light object in the no-switch condition (light-after-light; mean difference = 3.43, *t* = 6.06, *p* < .001).

#### Peak grip force (PGF)

A main effect of cube was found (*F*(1, 28) = 353.64, *p* <.001, *η*_*p*_^*2*^ =.93), with the heavy cube being lifted with a greater average peak grip force than the light cube (mean difference = 4.19, *t* = 18.81, *d* = 2.57, *p* < .001). There was also a Cube × Switch interaction (*F*(1, 28) = 18.95, *p* < .001, *η*_*p*_^*2*^ =.40; Fig. 4). Post hoc testing revealed that the heavy cube was lifted with a lower average peak grip force when it was lifted following the light cube in the switch condition (heavy-after-light) as compared to the heavy cube in the no-switch condition (heavy-after-heavy; mean difference = 0.31, *t* = 2.35, *d* = 0.19, *p* = .026). A similar effect was found for the light cube, where a greater average peak grip force was applied in the switch (light-after-heavy) as compared to no-switch trials (light-after-light; mean difference = 0.43, *t* = 6.41, *d* = 0.26, *p* < .001).

#### Load force at first peak in load force rate (LF_1st_)

There was a main effect of cube (*F*(1, 28) = 96.62, *p* < .001, *η*_*p*_^*2*^ = .78), with the heavy cube being lifted with a greater LF_1st_ on average than the light cube (mean difference = 0.63, *t* = 9.83, *d* = 2.08, *p* < .001). An interaction was also found between the cube and switch conditions (*F*(1, 28) = 6.78, *p* = .02, *η*_*p*_^*2*^ = .20; Fig. 4), with a lower average LF_1st_ applied to the heavy cube in the switch (heavy-after-light) as compared to the no-switch condition (heavy-after-heavy; mean difference = 0.13, *t* = 2.46, *d* = 0.41, *p* = .020). A similar effect was found for the light cube, being lifted with a greater average LF_1st_ in the switch (light-after-heavy) as compared to the no-switch condition (light-after-light; mean difference = 0.04, *t* = 2.20, *d* = 0.13, *p* = .036).

#### Load phase duration 1 (LPD1)

The ANOVA revealed a main effect of cube (*F*(1, 28) = 138.15, *p* < .001, *η*_*p*_^*2*^ = .83), where the heavy cube, on average, took longer to lift than the light cube (mean difference = 0.46, *t* = 11.75, *d* = 2.68, *p* < .001). There was an interaction between the cube and switch conditions (*F*(1, 28) = 20.13, *p* < .001, *η*_*p*_^*2*^ = .42; Fig. 4), with the heavy cube being lifted more slowly in the switch (heavy-after-light) as compared to no-switch condition (heavy-after-heavy; mean difference = 0.04, *t* = 5.67, *d* = 0.51, *p* < .001), while no such effect was found for the light cube (*p* =.526).

#### Peak platform force rate (PPFR)

There was the expected main effect of cube (*F*(1, 28) = 114.92, *p* < .001, *η*_*p*_^*2*^ = .80), where the heavy cube was lifted with a greater average peak platform force rate than was the light cube (mean difference = 13.62, *t* = 10.72, *d* = 1.57, *p* < .001). There was also an interaction between the two conditions (*F*(1, 28) = 11.06, *p* = .002 *η*_*p*_^*2*^ = .28; Fig. 4), with post hoc tests finding that the heavy object was lifted with less average force following a switch (heavy-after-light) as compared to no-switch condition (heavy-after-heavy; mean difference = 3.50, *t* = 3.03, *d* = 0.41, *p* = .005). There was no switch-dependent influence on the light object (*p* = .511).

#### Platform force at first peak in platform force rate (PF_1st_)

There was a main effect of cube (*F*(1, 28) = 114.89, *p* < .001, *η*_*p*_^*2*^ = .80), with a greater force rate recorded for the heavy as compared to the light cube (mean difference = 1.10, *t* = 10.72, *d* = 2.66, *p* < .001; Fig. 4). There was no Cube × Switch interaction (*p* = .121).

#### Load phase duration 2 (LPD2)

The ANOVA on the platform-based load phase duration data found a main effect of cube (*F*(1, 28) = 214.72, *p* < .001, *η*_*p*_^*2*^ = .89) in which the heavy cube took longer on average to lift than did the light cube (mean difference = 0.24, *t* = 14.65, *d* = 2.85, *p* < .001). There was also an interaction effect between the cube and switch conditions (*F*(1, 28) = 30.72, *p* < .001, *η*_*p*_^*2*^ = .52; Fig. 4), in which there was a longer LPD2 for the heavy cube in the switch as compared to the no-switch condition (mean difference = 0.04, *t* = 5.35, *d* = 0.52, *p* < .001), and the LPD2 for the light cube was longer when it was lifted in a switch versus no-switch condition (mean difference = 0.01, *t* = 3.27, *d* = 0.15, *p* = .003).

### Experiment 1: Intraclass correlations

Overall, the force data collected via the handles and platform in Experiment 1 demonstrated moderate-to-excellent agreement in both peak (PLFR and PPFR) and force at first peak (LF_1st_ and PF_1st_) measures for the heavy object. However, for the light object, the agreement was lower, ranging from poor to good. A similar pattern was observed in the LPD1 versus LPD2 comparisons, with stronger agreement between measures for the heavy than the light condition. A summary of this information is presented in Table 1. As discussed later, we propose that this disparity arises from the platform’s limited capacity to record data, suggesting a meaningful difference relevant to their use.

**Table 1:** Intraclass correlations between handle and platform measures in Experiment 1

Comparison	ICC(3, 1)	95% CI
*Force rate peak data*		
Heavy-after-heavy	.87	[.74 to .94]
Heavy-after-light	.83	[.66 to .92]
Light-after-light	.80	[.62 to .90]
Light-after-heavy	.71	[.47 to .85]
*Force at first peak in force rate*		
Heavy-after-heavy	.87	[.75 to .94]
Heavy-after-light	.87	[.73 to .93]
Light-after-light	.65	[.38 to .82]
Light-after-heavy	.68	[.42 to .83]
*Load phase duration*		
Heavy-after-heavy	.98	[.96 to .99]
Heavy-after-light	.98	[.96 to .99]
Light-after-light	.76	[.55 to .88]
Light-after-heavy	.49	[.16 to .72]

An ICC value of < .50 corresponds to poor agreement, .50 – .75 corresponds to moderate, .75 – .90 to good, and > .90 to excellent (Koo & Li, 2016)

#### LFRP vs PPFR

We compared the force data collected simultaneously from the handles and platform in Experiment 1 to assess the concurrent validity of the measures. Specifically, for the heavy-after-heavy trials, the ICC(3, 1) was .87 with a 95% CI between .74 and .94, indicating moderate-to-excellent agreement between measures. For the heavy-after-light data, the ICC(3, 1) was similar, with an estimate of .83, with a 95% CI between .66 and .92, again showing moderate-to-excellent agreement between measures. Regarding the light-after-light data, the ICC(3, 1) was .80 with a 95% CI between .62 and .90, indicating moderate-to-excellent agreement between the handle and platform measures. Finally, the ICC(3, 1) of the light-after-heavy data had an estimate of .71 with a 95% CI between .47 and .85, representing poor-to-good agreement, notably lower than the other three conditions.

#### LF_1st_ vs PF_1st_

We ran similar ICCs on the force at first peak in force rate data recorded from both the handles (LF_1st_) and the platform (PF_1st_). For the heavy-after-heavy data, the ICC(3, 1) was .87, with a 95% CI between .75 and .94, and that of the heavy-after-light data was .86 with a 95% CI between .73 and .93, showing good-to-excellent and moderate-to-excellent agreement between measures, respectively. Regarding the light cube, the light-after-light data between the two recoding methods had an ICC(3, 1) of .65 with a 95% CI between .38 and .82, indicating moderate-to-good agreement. Finally, the light-after-heavy data had an ICC(3, 1) of .68 with a 95% CI between .42 and .83, representing poor-to-good agreement. Overall, stronger agreement between measures was seen for the heavy as compared to the light condition.

#### LPD1 vs LPD2

Similar ICCs were also conducted for the load phase duration data produced from the handles (LPD1) and the platform (LPD2). For the heavy-after-heavy trials, there was excellent agreement between the two load phase measures (ICC(3, 1) = .98, 95% CI [.96 to .99]). A similar level of agreement was observed for the heavy-after-light data, with an ICC(3, 1) of .98 (95% CI [.96 to .99]). Regarding the light object, the extent of agreement between measures was not as strong as with the heavy, with the light-after-light data having an ICC(3, 1) of .76, with a 95% CI between .55 and .88 — moderate-to-good agreement. For the light-after-heavy data, the ICC(3, 1) was.49 (95% CI [.16 to .72]) representing poor-to-moderate agreement between measures.

### Experiment 2: Lifting objects directly

**Fig. 5:**
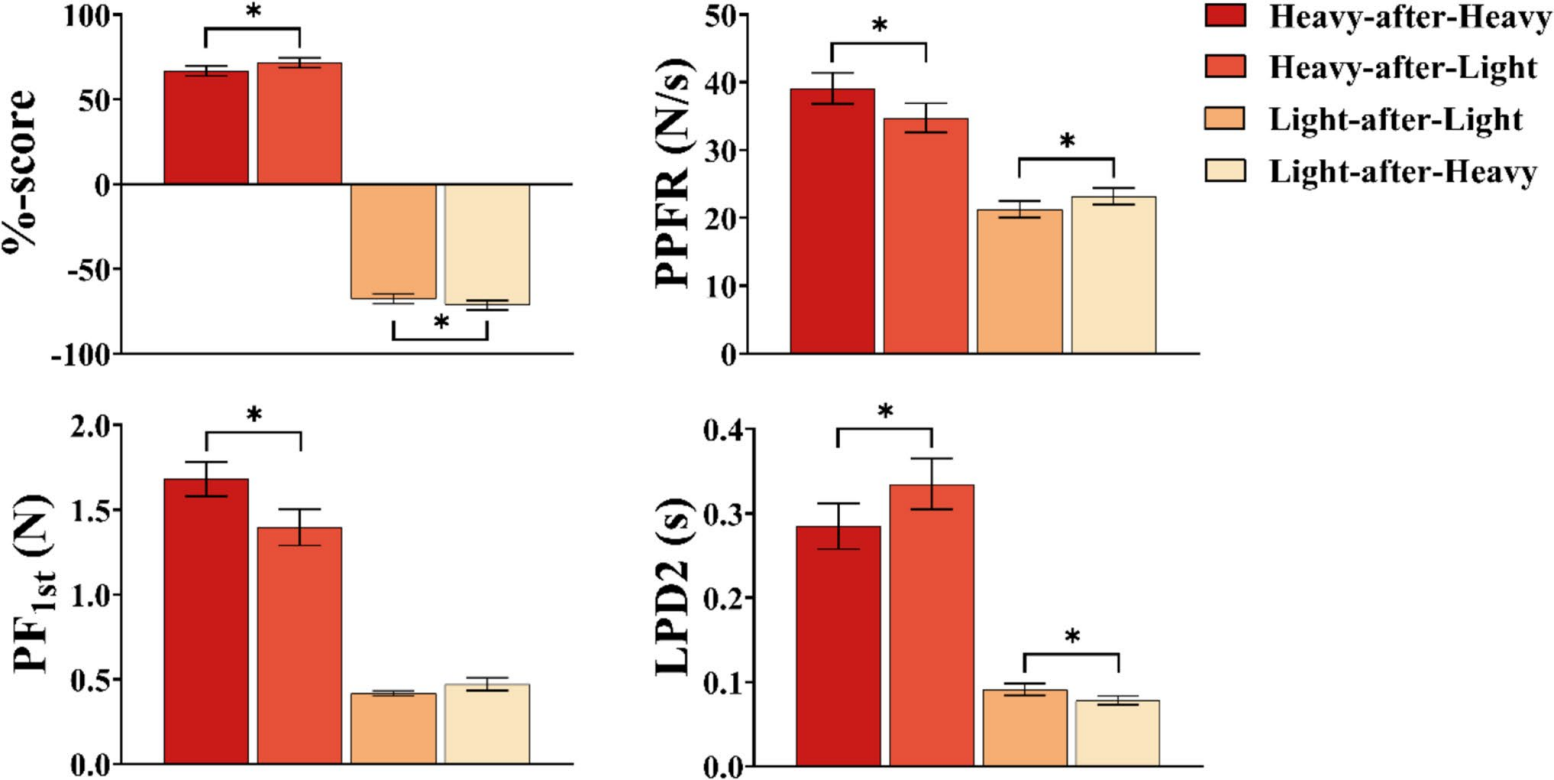
ANOVA results for dependent variables in Experiment 2. Displayed are means with error bars representing ±1 standard error of the mean for %-scores, peak platform force rate (PPFR), platform force at first peak in platform force rate (PF_1st_), and load phase duration 2 (LPD2). Only comparisons of interest are displayed (i.e., switch vs no-switch for the heavy and light cubes). Asterisks (*) denote a significant effect (*p* < .05)

Overall, we found strong and consistent interaction effects across perception, PPFR, and LPD2 for both the heavy and light cubes, where the heavy cube was perceived as lighter and lifted with less force when it followed a light cube, and the light cube was perceived as heavier and lifted with more force when it followed a heavy cube. However, interaction effects for PF_1st_ were observed only for the heavy cube. A summary of the ANOVA outcomes is provided in Table 2, with individual variables presented in Fig. 5.

**Table 2:** ANOVA summary for interaction effects of interest in both experiments

	Measure	Heavy		Light	
		*p*	*d*	*p*	*d*
Exp 1	Perception	.047	0.148	< .001	0.318
	PLFR	< .001	0.452	.001	0.199
	PLF	.059	0.186	.018	0.117
	PGFR	< .001	0.430	< .001	0.362
	PGF	.026	0.189	< .001	0.261
	LF_1st_	.020	0.409	.036	0.130
	LPD1	< .001	0.514	.526	0.044
	PPFR	< .005	0.405	.511	0.069
	PF_1st_	–	–	–	–
	LPD2	< .001	0.517	.003	0.151
Exp 2	Perception	.004	0.301	< .001	0.236
	PPFR	< .001	0.450	< .001	0.196
	PF_1st_	< .001	0.690	.127	0.131
	LPD2	< .001	0.449	< .001	0.115

Comparisons reported in the heavy and light columns comprise switch versus no-switch conditions. Dashes (–) indicate no significant interaction effect

#### Perception (%-scores)

The ANOVA revealed a main effect of cube (*F*(1, 28) = 603.11, *p* < .001, *η*_*p*_^*2*^ = .96), in line with the mass of the stimuli: the heavy cube was perceived as being heavier than the light cube (mean difference = 138.66, *t* = 24.56, *d* = 8.91, *p* < .001). The was also a Cube × Switch interaction (*F*(1, 28) = 18.13, *p* < .001, *η*_*p*_^*2*^ = .39), where the heavy cube was perceived as being heavier when it was lifted after the light (heavy-after-light) versus the heavy cube (heavy-after-heavy; mean difference = 4.68, *t* = 3.11, *d* = 0.30, *p* = .004), and the light cube was perceived as lighter following the heavy (light-after-heavy) versus the light cube (light-after-light; mean difference = 3.68, *t* = 4.60, *d* = 0.24, *p* < .001).

#### Peak platform force rate (PPFR)

A main effect of cube was found (*F*(1, 28) = 147.75, *p* < .001, *η*_*p*_^*2*^ = .84), indicating that the platform recorded a greater peak in lifting force on average when participants lifted the heavy versus light cubes (mean difference = 14.72, *t* = 12.16, *d* = 1.52, *p* < .001). There was also a Cube × Switch interaction (*F*(1, 28) = 54.89, *p* < .001, *η*_*p*_^*2*^ = .66; Fig. 5), where the platform recorded a lower average peak force rate when the heavy cube was lifted in the switch (heavy-after-light) than in the no-switch condition (heavy-after-heavy; mean difference = 4.37, *t* = 6.91, *d* = 0.45, *p* < .001). Post hoc testing also revealed that the light object was lifted with greater force in the switch (light-after-heavy) versus no-switch (light-after-light) condition (mean difference = 1.90, *t* = 4.03, *d* = 0.20, *p* < .001).

#### Platform force at first peak in platform force rate (PF_1st_)

There was a main effect of cube (*F*(1, 28) = 114.89, *p* < .001, *η*_*p*_^*2*^ = .80), with greater PF_1st_ being applied to lift the heavy than the light cube (mean difference = 1.10, *t* = 10.72, *d* = 2.66, *p* < .001). There was a Cube × Switch interaction effect (*F*(1, 28) = 12.67, *p* = .001, *η*_*p*_^*2*^ = .31; Fig. 5), with post hoc tests indicating that a lower PF_1st_ was recorded when the heavy cube was lifted in the switch (heavy-after-light) as compared to the no-switch condition (heavy-after-heavy; mean difference = 0.28, *t* = 3.98, *d* = 0.69, *p* < .001). A similar influence of switch was not significant for the light cube, however (*p* = .127).

#### Load phase duration 2 (LPD2)

The ANOVA on the platform-based load phase duration measure revealed a main effect of cube (*F*(1, 28) = 95.40, *p* < .001, *η*_*p*_^*2*^ = .77), indicating that the load phase duration was on average 0.23 s longer for the heavy than for the light cube (*t* = 9.77, *d* = 2.02, *p* < .001). There was also a Cube × Switch interaction (*F*(1, 28) = 55.56, *p* < .001, *η*_*p*_^*2*^ = .67; Fig. 5), with post hoc testing indicating that, on average, participants took longer to lift the heavy cube in the switch (heavy-after-light) than in the no-switch (heavy-after-heavy) condition (mean difference =.05, *t* = 7.33, *d* = 0.45, *p* < .001). Post hoc testing also revealed that the light object was lifted faster in the switch (light-after-heavy) than the no-switch (light-after-heavy) condition (mean difference = 0.01, *t* = 4.33, *d* = 0.12, *p* < .001).

## Discussion

This study aimed to investigate whether a force transducer-embedded platform can serve as a viable alternative to force transducer-embedded handles in object-lifting tasks, specifically in terms of data validity and accuracy, and the experimental outcomes they produce. To this end, we evaluated the platform from two perspectives: first, by examining the degree to which its force data aligned with the data recorded by handles during lifting (concurrent validity, via Experiment 1), and second, by assessing whether it reproduced the same experimental outcomes as the handle-based method when used in its typical form (convergent validity — that is, by comparing Experiment 1 and Experiment 2). In the following discussion, we highlight two major findings. First, when used in its typical manner, the experimental outcomes produced by the platform broadly align with those of the handle, supporting its convergent validity. Second, when data were collected simultaneously, the platform-based method demonstrated robust concurrent validity with the handle-based method for heavy objects, though this was less robust for the light objects.

### Experimental outcomes of the platform compared to handles

To evaluate the performance of the platform-based method relative to the established handle-based approach, we analysed the experimental outcomes from both methods during typical object-lifting tasks. The results of Experiment 1, in which objects were lifted using handles, strongly aligned with expectations (e.g., Harris et al., 2024; van Polanen & Davare, 2015). Participants’ perception of the objects’ weights was influenced by preceding lifts, with lighter objects feeling even lighter when following a heavier object, and vice versa. Similar sensorimotor effects were evident in force application, as peak load force rates, peak grip force rates, and peak grip forces each demonstrated switch influences for both objects. Switch effects were also found in peak load forces, but only for the light object. The load force at first peak also showed the expected switch influence, indicating that participants’ anticipatory force scaling was affected by preceding lifts. Finally, in the handle-based load phase measure, switch effects were found for the heavy, but not the light, object. Overall, 12 out of the 14 switch comparisons revealed the anticipated sensorimotor effects, strongly supporting findings from past research (Chouinard et al., 2005; Harris et al., 2024; Johansson & Westling, 1988; van Polanen & Davare, 2015), and consistent with assimilation effects widely observed in psychophysical tasks, where responses are systematically biased toward the preceding stimulus (Stewart et al., 2005).

In Experiment 2, where the objects were lifted directly from the platform, the results again aligned with our expectations. Participants’ perception of the object’s weight depended on the switch condition, with the light object feeling lighter and the heavy object feeling heavier after a switch. Peak platform force rates mirrored these switch effects, with greater force applied to the light object and less to the heavy object after a switch. Interestingly, peak force rate measures are, to our knowledge, not reported in studies using the platform, which instead focus on force at first peak measures. This is important, as peak and first peak measures may reflect different aspects of the sensorimotor process. Our findings suggest that the platform can effectively capture peak force rates, broadening its potential application.

In terms of the force at first peak in the platform force rate (PF_1st_), less force was applied to the heavy object when it followed the light, aligning with LF_1st_ findings from Experiment 1 and handle-based findings from previous work (van Polanen et al., 2022). While an interaction was not seen for the light object, it has been common practice for its data to be removed from analysis and interpretation in previous platform-based studies (Baugh et al., 2016; Flanagan et al., 2008; Trewartha & Flanagan, 2017) due to an inherent limitation of the platform in that it can only record force data up until the point of lift-off. In cases where lift-off occurs rapidly — such as when a light object is lifted with excessive load force — the first peak in load force rate may occur after lift-off, and beyond the platform’s recording capacity. Nonetheless, Experiment 2 demonstrates that the switch manipulation influenced perception, load phase, and lifting forces, all in a manner which aligns with sensorimotor expectations, and importantly, with Experiment 1.

However, it is worth noting the platform’s limitations. Not only is it unable to record grip forces, but the force measure typically derived from its use, PF1st, may be unreliable for up to half of the data — specifically, the trials involving the light object — due to the platform’s inability to capture force beyond the point of lift-off. Consequently, the platform may effectively have only one-eighth of the usable data compared to the handle-based measures. With fewer data points, the platform could give the impression of more consistent outcomes, potentially limiting its ability to capture nuanced differences in force application.

Ultimately, despite these limitations, when considered in isolation, it is clear that the platform-based method led to the same overarching conclusions regarding sensorimotor influences as the handle-based method, serving as strong evidence for the convergent validity of both methodologies. The convergence not only reinforces the consistency and reliability of sensorimotor effects but also underscores the platform’s potential as a valid alternative for studying these influences, even when it cannot provide data with the level of detail possible using handle-based methods.

### Comparison of platform- and handle-derived data

 We also sought to assess the validity of the platform method by comparing the data it recorded to that recorded simultaneously via the handle. For the peak load / platform force rate data, we found moderate to excellent agreement across both switch and no-switch conditions for the heavy object. However, for the light object, the level of agreement was notably lower, ranging from moderate to good for light-after-light conditions and from poor to good for light-after-heavy conditions. A similar pattern emerged in the force at first peak data, with agreement for the heavy object being moderate-to-excellent and good-to-excellent for the no-switch and switch conditions, respectively. For the light object, however, agreement was again reduced, being moderate-to-good regardless of the switch condition. This pattern continued in the load phase duration data. Specifically, we observed excellent agreement in both LPD1 and LPD2 for the heavy object under both switch and no-switch conditions, indicating that both the platform and handles identified the point of lift-off with high consistency. For the light object, however, agreement ranged from moderate to good for no-switch lifts and from poor to moderate for switch conditions. Across all measures — peak force, first peak force, and load phase duration — greater consistency was found for the heavy object compared to the light object.

The disparity in agreement between methods depending on object weight is notable, and its influence can be observed in the experimental outcomes of the ANOVA in Experiment 1. For example, in the handle-based peak force measure, significant interactions were found for both object weights, whereas the platform-based data only showed interactions for the heavy object. In the first peak force measure, the handle-based data showed the expected interactions for both object weights, but no interaction effect was observed for the platform-based measure. A similar method-based difference emerged in the load phase measure, where interactions were significant only for the heavy object using handles, but for both heavy and light objects with the platform. This highlights the potential problems of assuming comparability between handle and platform data, particularly for light objects, as incorrect assumptions could lead to substantially different experimental outcomes in Experiment 1.

Overall, the intraclass correlation analysis suggests that the platform-based method demonstrates robust concurrent validity with the handle-based method for heavy object data. However, for the light object, agreement was consistently lower across force and timing measures. Notably, this discrepancy cannot be attributed to differences in perceived weight based on expectation mismatch, as these effects were observed for both the heavy and light objects. Yet, this consistency was not mirrored in the ICCs, where divergence emerged only for the light object. This suggests that the observed differences are likely due to limitations in recording force for lower-mass objects using the platform — rather than expectation-based influences — and may reflect mass-dependent differences in how the platform captures force at or near lift-off. While our design included only two object masses, these findings indicate that the platform may be less reliable for measuring forces when object mass is low. Accordingly, future studies using the platform should either consider excluding lighter object data, as done in prior work (Baugh et al., 2016; Flanagan et al., 2008; Trewartha & Flanagan, 2016), or interpret such data with caution. Although many questions can still be addressed using heavier objects alone, investigations specifically targeting lighter weight ranges may require alternative methods, such as the traditional handle-based approach.

### Concluding remarks

 While the platform method has limitations — including an inability to measure grip forces and reduced data capture for lighter objects — our findings help clarify how it compares to the established handle-based approach. Specifically, we found evidence of both convergent and concurrent validity for heavy objects, providing confidence in the comparability of findings across methods and measures under these conditions. However, the same level of consistency was not observed for the light object, suggesting that the two methods should not be considered interchangeable in all contexts. These results offer a more informed basis for methodological decision-making, supporting the platform’s use in studies where handle-based methods are impractical or where naturalistic interactions are prioritised, while also highlighting the need for caution when applying it in other settings. Future research may better identify the specific ranges of object weights that are best suited for the platform method to further clarify its optimal use.

## Data Availability

Data publicly available via OSF: https://osf.io/2j8gv/.

## Declarations

### Preregistration

The experiments here were not preregistered.
